# A simple method for inducing nonalcoholic steatohepatitis with fibrosis

**DOI:** 10.1002/ame2.12089

**Published:** 2019-11-14

**Authors:** Leyla Yahaghi, Azadeh Ebrahim‐Habibi, Nasim Hayati‐Roodbari, Shiva Irani, Parichehreh Yaghmaei

**Affiliations:** ^1^ Department of Biology, Science and Research Branch Islamic Azad University Tehran Iran; ^2^ Biosensor Research Center Endocrinology and Metabolism Molecular‐Cellular Sciences Institute Tehran University of Medical Sciences Tehran Iran; ^3^ Endocrinology and Metabolism Research Center Tehran University of Medical Sciences Tehran Iran

**Keywords:** animal model, nonalcoholic fatty liver disease (NAFLD), nonalcoholic steatohepatitis (NASH), PPAR‐α

## Abstract

**Background:**

Nonalcoholic fatty liver disease (NAFLD) is increasingly occurring in sedentary people, and may progress to NASH and hepatocellular carcinoma. It is essential to design affordable animal models for the study of various diseases, including fatty liver, which was the aim of the study. In this study, a high‐fat diet was devised that triggers NASH's animal model quickly and easily. High‐fat diet (HFD) was used both with intra‐mouth oral gavage and in combination with animal pellets.

**Methods:**

Twenty‐four male C57BL/6J mice were divided into HFD and ND groups, which received a high‐fat diet and a normal diet, respectively. At the end of the experiment (fourth week of treatment), body and liver weights, biochemical parameters, PPAR‐α gene expression and histopathologic characteristics of the liver were evaluated.

**Results:**

During 4 weeks, body weight of mice did not show a significant increase in the HFD group compared to the ND group, while weight gain of the liver was significant. Histological assessment of the HFD group's liver confirmed NASH symptoms. In the HFD group, HDL‐c, SOD, catalase, FRAP, adiponectin, and PPAR‐α decreased significantly, and lipid profiles, hepatic enzymes, MDA, leptin, and TNF‐α showed a significant increase compared to the ND group.

**Conclusion:**

Our high‐fat diet has successfully induced all aspects of NASH with fibrosis in 4 weeks, and with low cost.

## 1 INTRODUCTION

The main risk factor for many diseases, such as overweight, obesity, atherosclerosis, hypertension, myocardial infarction, and NAFLD is hypercholesterolemia. NAFLD is increasingly observed in sedentary people and is prevalent in patients with high blood cholesterol levels. NAFLD is induced by increased triglyceride accumulation leading to steatosis, and this may progress to NASH, steatofibrosis, and subsequently lead to cirrhosis and hepatocellular carcinoma.^1^ NAFLD is accompanied by enhanced cardiovascular risk factors. Furthermore, atherosclerotic symptoms usually include carotid plaques, and coronary arterial calcification occurs in NAFLD patients.^2^ NAFLD is one of the important occurrences of liver diseases in developed countries. This type of fatty liver is related to lifestyle and may worsen to cirrhosis and hepatocellular carcinoma.^3^

Appropriate animal models are essential for research on various diseases and for finding suitable therapies for them.^4^ The use of animal models is particularly useful for biological studies like assessing the effect of different dietary patterns on various diseases and metabolic phenotypes. Close similarities in genetics and environmental status play a key role in experimental animal model's research for extrapolation to human subjects.^5^ Although animal models exist for NAFLD, there are a few models that have similarities with basic characteristics of fatty liver's human. Some experimental animal models that are used for dyslipidemia, obesity, and steatohepatitis studies are valuable for nonalcoholic steatohepatitis (NASH) investigations.^4^ Moreover, some animal models are used for the assessment of steatohepatitis and steatosis, but these models do not provide identical results regularly.^6^ The diets used to induce the formation of cholesterol crystals and gallstones were HFD containing between 200% and 500% of the fat present in the regular chow diet. Not unexpectedly, most of the experimental animals developed fatty livers.^7^

HFD was used for rats by Zou and co‐workers as stomach gavage for 6 weeks.^4^ Chao‐Yung Wang and James K. Liao used an HFD of 60 kcal% in different conditions for 16‐20 weeks.^8^ A HFD was used that contains 2% cholesterol and 0.5% cholic acid in rats for 12 weeks.^2^ Various HFD has been used in different experimental animal models with almost 20%‐60% energy from lipid, and its derivatives. Fat sources of these HFD were plants and animal lipids. Some researchers have designed HFD either by replacing carbohydrates with fats or by providing a standard chow and fat mixture or using carbon tetrachloride (a toxic compound) as an accelerator for NASH animal model induction.^9^, ^10^ The goal of this research was to design a HFD regime that would be inexpensive, quick to show results, and closely resemble the human obese diet and western lifestyle to ultimately trigger NASH in C57BL/6J mice model.

## 2 MATERIALS AND METHODS

All compounds were purchased from Sigma‐Aldrich unless otherwise stated.

### 2.1 HFD preparation for gavage

This diet was prepared by fats (562.89 calories/100 mL), carbohydrates (33.63 calories/100 mL), and protein (1.04 calories/100 mL). The mice in the HFD group were gavaged by 12 mL/kg/d of HFD for 4 weeks. These ingredients and calculation of the energy values of ingredients are stated in Table 1.^11^ The resulting emulsion was well‐mixed and filtered by mesh size of 0.354 mm, then aliquoted in appropriate volumes, and stored at 4°C. The stored emulsion was incubated at 37°C for 10 minutes before administration.

**Table 1:** Ingredients of oral gavage diet

Ingredient	Composition of oral gavage diet (%)
Sunflower oil	59.3
Cholesterol	3.5
Milk powder (Aptamil 3 Co.)	10
Sucrose	4
Fructose	4
Sodium deoxycholate	0.35
Tween 80	1.3
Propylene glycol	1.1
Multivitamin (Wellkid, UK)	0.1
NaCl	0.35
Distilled water	16

### 2.2 HFD preparation for feeding of ad libitum

The HFD was mixed with standard chew pellet by a ratio of 1:5. For ad libitum feeding, HFD was prepared by adding a mixture of fats (774 calories/100 mL of HFD), milk powder as protein portion (1.456 calories/100 mL of HFD), and carbohydrate (5.04 calories/100 mL of HFD) to standard rodent chow.

This HFD emulsion was administered ad libitum for animals that received HFD compounds as gavage simultaneously. The ingredients of ad libitum HFD‐feeding have been stated in Table 2.

**Table 2:** Ingredients of ad libitum HFD‐feeding

Ingredient	Percentage (%)
Sunflower oil (Bahar, Iran)	50
Palm oil shortening (Malaysia)	16
Hydrogenated vegetable oil (Bahar, Iran)	15
Cholesterol	5
Milk powder	14

### 2.3 Animals

Twenty‐four male C57BL/6Jmice (25 ± 2 g, 6 weeks old) were purchased from the Pasteur Institute, and housed in dry, clean, and appropriate cages, in an animal room that was well air‐conditioned at 25°C, and under 12 hours light and 12 hours dark period.

Experimental design: the animals were weighed and randomly divided into two groups (n = 12). After 1‐week animal adaptation with regular conditions and foods, ND (normal diet) group received standard diet for 4 weeks, and HFD group received 12 mL/kg/d HFD for 4 weeks. All mice were weighed weekly. After 4 weeks of treatment, mice were anesthetized with ketamine‐xylazine, and their blood was collected from the hearts. Then the livers were removed and labeled for histological and biochemical experiments. This protocol was confirmed by the animal committee of the science and research branch, Azad University, Tehran and international guidelines provided for guide care and use of laboratory animals.

### 2.4 Histological and biochemical assessment

Liver tissues were fixed in 10% formalin immediately after being removed from mice. The tissues were stained with hematoxylin and eosin (H&E) dye for the detection of NASH conditions and Masson's trichrome stain for liver tissue fibrosis detection. The stained sections were observed under a light microscope. Steatohepatitis was evaluated by histopathological analysis of the liver slices.

The livers (1:3 w/v) were homogenized in 50 mM phosphate‐buffered saline (pH = 7.4) and sonicated for 1.5 minutes. All processes were carried out at 0‐4°C. Homogenized samples were centrifuged at 22 000*g* for 17 minutes at 4°C. The supernatant was stored at 40°C until analysis. Liver tissues were used for biochemical assessments including liver catalase (CAT), liver superoxide dismutase (SOD), liver homogenate malondialdehyde (MDA) by Goth, Makland, and Buege methods, respectively.^12^, ^13^, ^14^ Furthermore, total cholesterol (TC) and triglyceride (TG) were measured according to the ZistChimi kit's protocol (ZistChimi Chemical Company). The total protein (TP) was evaluated according to the biuret method.^15^

Quantitative determination of the total lipid (TL) was performed by using the sulfo‐phospho‐vanillin colorimetric method.^16^, ^17^ Phospholipid (PL) assessment was performed according to the manufacturer's protocol (EnzyChromTM).

Blood serum was used for the assessment of aspartate aminotransferase (AST), alanine aminotransferase (ALT), alkaline aminotransferase (ALP), high‐density lipoprotein cholesterol (HDL‐C), low‐density lipoprotein cholesterol (LDL‐C), and glucose according to ZistChimi kit's protocol (ZistChimi Chemical Company). Ferric reducing antioxidant power (FRAP) was evaluated according to the Koracevic protocol.^18^ Insulin assessment was performed by mouse ultrasensitive insulin ELISA kit (ALPCO Diagnostics). The insulin resistance index, homeostasis model assessment (HOMA), was determined by this formula: homeostasis model assessment = fasting serum insulin (mU/L) × fasting plasma glucose (mM)/22.5.^19^ The atherogenic index was calculated by (total cholesterol ‐HDL‐C)/(HDL‐C) formula.^20^ The level of cytokine tumor necrosis factor‐alpha (TNF‐α) in serum was analyzed by the mouse TNF‐alpha ELISA Kit eBioscience, USA. Serum adiponectin and leptin measurement kits (mouse adiponectin, ADP ELISA kit; and mouse leptin, LEP ELISA kit) were used from Yanaihara Institute Inc, Japan and Otsuka Pharmaceutical Co., Japan, respectively.

### 2.5 PPAR‐α expression

Total RNA was extracted from liver tissues according to the High Pure RNA Isolation Kit protocol (Roche). The extracted RNA was stored at −80°C until analysis. The NCBI database was used to obtain potential primer sequences of PPAR‐α and HPRT (used as a housekeeping gene). The experimental primers were designed by the Primer Express program. Thermo Scientific kit method and YTA SYBR Green qPCR MasterMix 2X protocol (YektaTajhizAzma) were used for the synthesis of cDNA and real‐time quantitative PCR, respectively. These primer sequences were used: 5′‐GGGGAACTTAGAGGAGAGCCAAG‐3′ as a forward, 5′‐CGCTAAGCTGTGATGACAACG‐3′ as the reverse for PPAR‐α gene, 5′‐TCAGACTGAAGAGCTACTGTAATGATCAG3′ as a forward and 5′‐TCAACAATCAAGACATTCTTTCCAG‐3′ as a reverse for HPRT gene.

### 2.6 Statistical analysis

The results were expressed as mean ± SEM. Differences between the ND group and HFD group were estimated via paired samples *t* tests after validating normality. Levels of statistical significance were considered at *P* < .05, *P* < .01, and *P* < .001.

## 3 RESULTS

### 3.1 Body and liver weight changes

**Figure 1:**
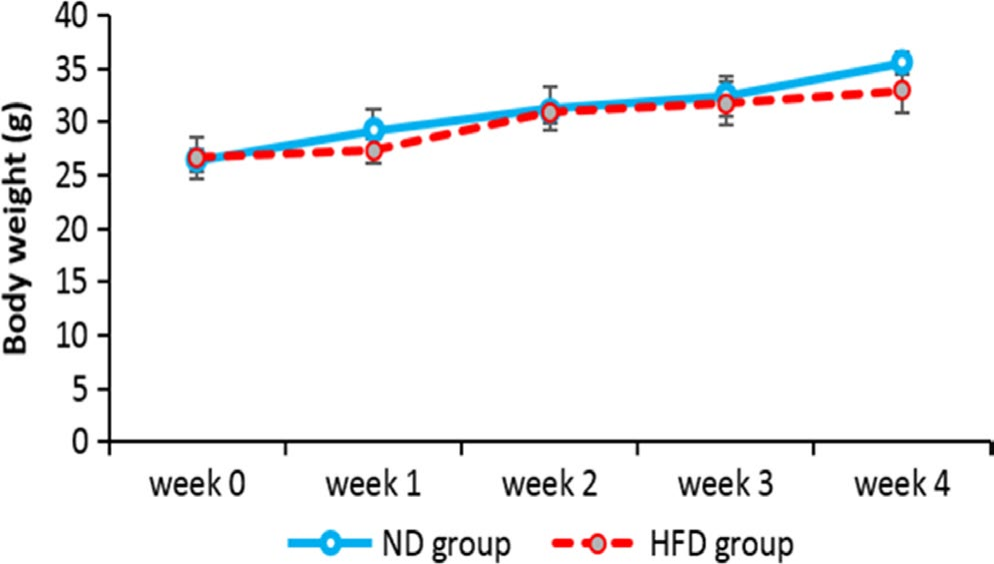
Effect of HFD on body weight in the ND and HFD groups during 4 weeks of treatment. The data were expressed as means ± SEM. There were no significant body weight differences between the ND and HFD groups. Abbreviations: HFD, high‐fat diet group; ND, normal diet group

According to Figure 1, there was no marked difference in the body weight of HFD compared with the ND group in the experiment duration. However, the liver weight index (%) ((liver weight/ body weight) × 100) showed a significant increase in the HFD compared with the ND group (*P* < .01) (Table 3).

**Table 3:** Liver weight index (%) changes in the ND and HFD mice groups after 4 weeks of trial

Parameters	ND group	HFD group
Liver weight index %	5.57 ± 0.53	8.14 ± 0.39**

Note

The data are expressed as Mean ± SEM.

Abbreviations: HFD, high‐fat diet group; ND, normal diet group.

** *P* < .01 compared with the control group.

### 3.2 Histological assessment

**Figure 2:**
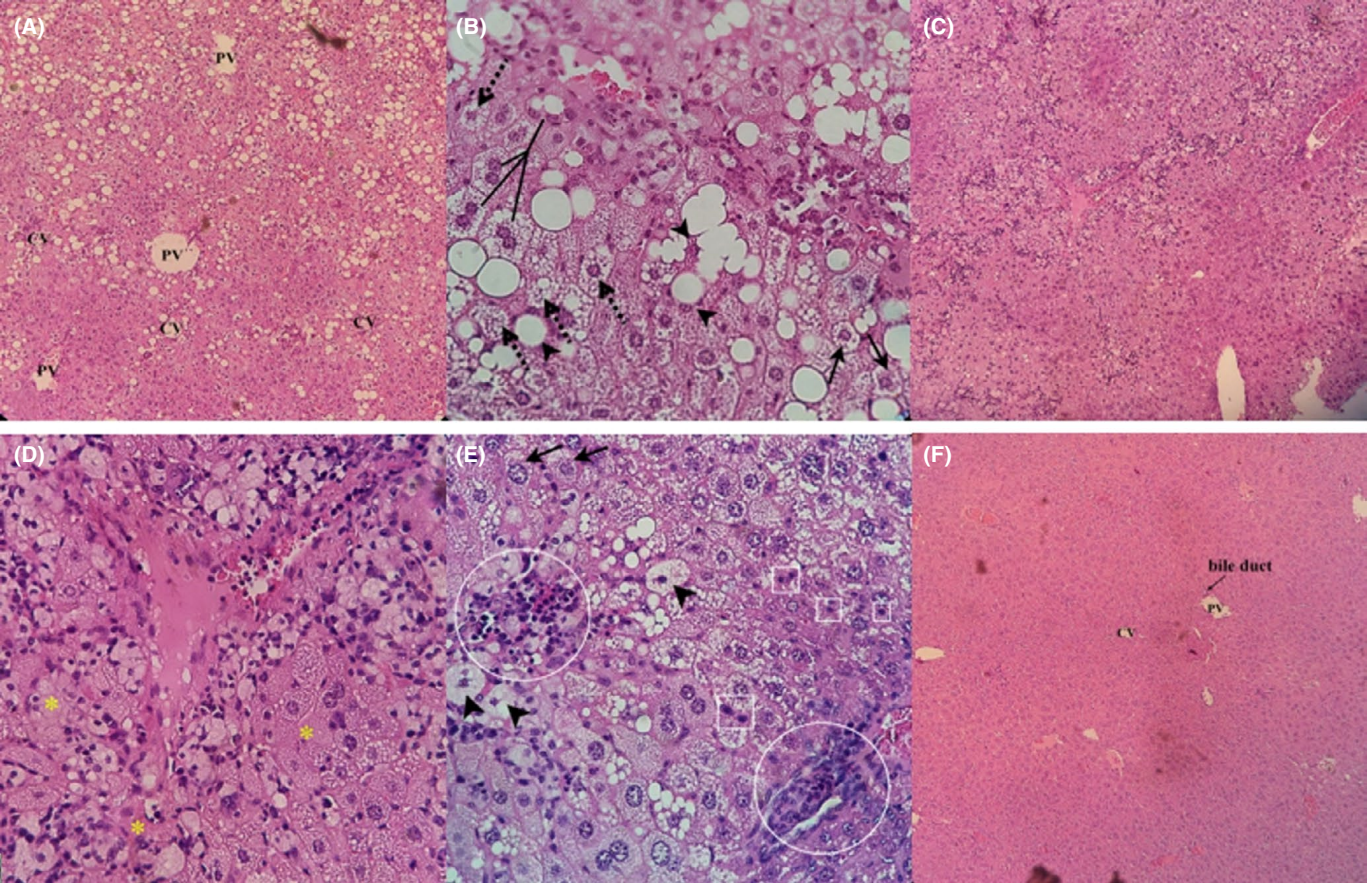
The liver sections of mice fed by HFD and ND after 4 weeks of trial. Liver hematoxylin and eosin staining; (A) steatosis, small and large lipid droplets (×160); (B) bipolar and tripolar cells (lines), foamy cytoplasm (dotted arrows), ballooned hepatocytes with the nuclei pushed to the border of cells (arrowheads), and MDB (arrows) (×640), (C, D) disruption of hepatocytes and necrosis (*), infiltration of mononuclear inflammatory cells (×160 and ×640, respectively); (E) MDB (arrows), ballooning (arrowheads), degenerative changes in hepatocytes, apoptotic cells (square), and inflammatory cell accumulation (circles) (×640); (F) normal liver (×160). Abbreviations: CV, central vein; MDB, Mallory‐Denk body; PV, portal vein

Steatohepatitis was confirmed by the analysis of liver tissue slices after 4 weeks of treatment (Figure 2). Fat accumulation and lipid droplets as micro‐ and macrovascular, bipolar, and tripolar cells, foamy cytoplasm, and hepatocyte's nuclei located peripherally in cell were found in the liver sections of HFD group (Figure 2A). In HFD group, ballooned hepatocytes, nuclei pushed to border of cells, disruption of hepatocytes and necrotic cells, and infiltration of mononuclear inflammatory cells were seen (Figure 2C). In the liver sections of HFD group, small and large droplets, periphery and hyperchromatic nuclei, foamy cytoplasm, ballooned hepatocytes, apoptotic cells (small dark with hypereosinophilic cytoplasm and pyknotic/ fragmented nuclei), degenerating cells, Mallory‐Denk body (MDB), and inflammatory cell clusters were observed (Figure 2E).

**Figure 3:**
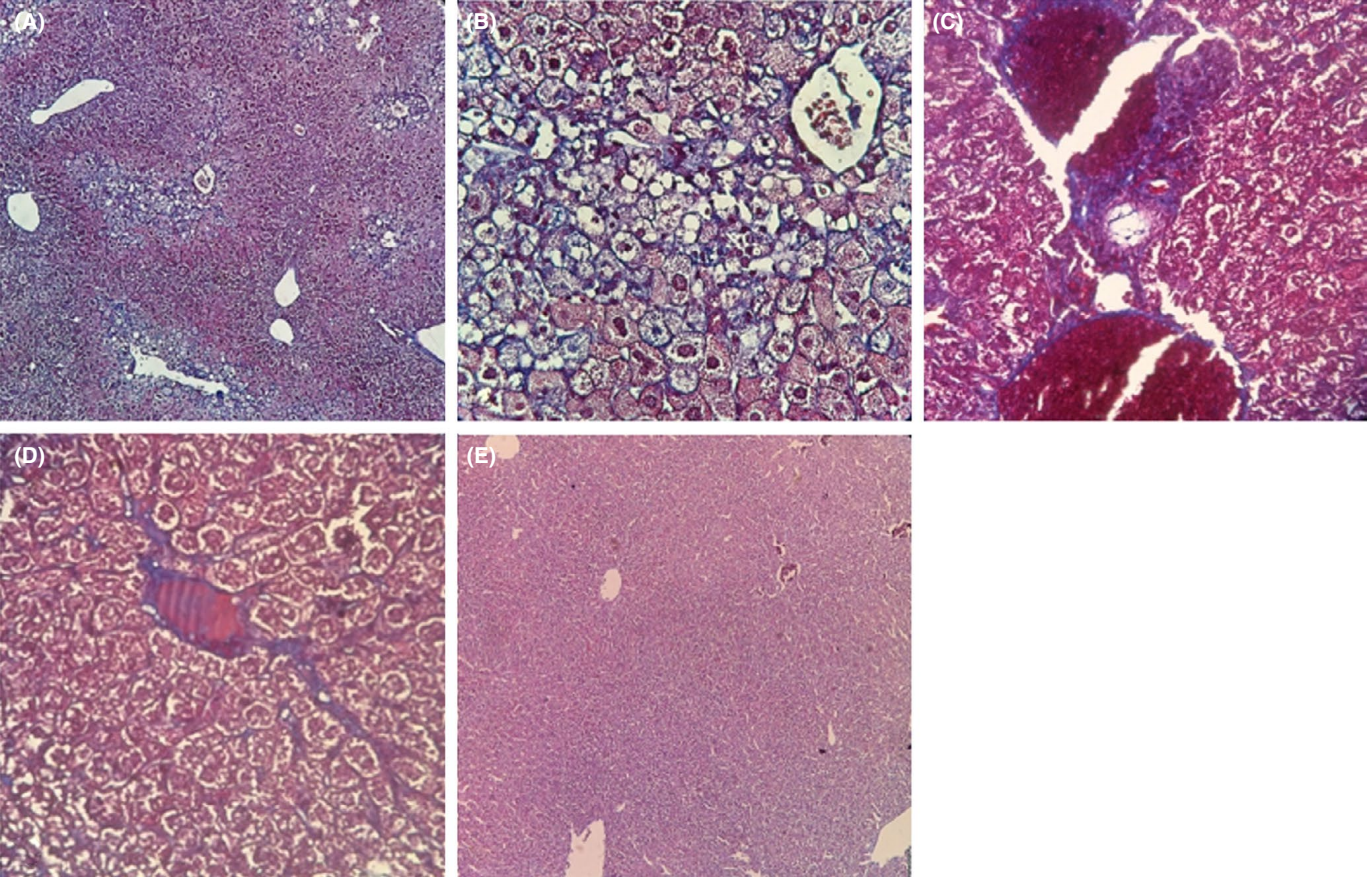
The livers of mice fed by HFD and ND after 4 weeks of trial. Liver Masson's trichrome staining; (A, B) bipolar and tripolar cells, lipid droplets, the nuclei pushed to the border of cells, foamy cytoplasm, ballooning, MDB, and pericellular/ perisinusoidal/ periportal fibrosis (×160 and ×640, respectively); (C) blood capillaries surrounded with fibrotic tissue (×640); (D) extension of fibrotic tissue from portal area to sinusoidal space (×640); (E) normal liver (×160)

The trichrome stain showed fibrosis development due to the collagen deposition around the portal area, sinusoidal spaces, and between hepatocytes as well as presence of MDB in hepatocytes and also, bipolar and tripolar cells, ballooning, nuclei pushed to the border of cells, and foamy cytoplasm (Figure 3).

### 3.3 Biochemical evaluation

Lipid profile, TP, fasting blood glucose (FBG), insulin, HOMA, and atherogenic index are shown in Table 4. Lipid levels were increased and HDL‐C was decreased significantly in HFD‐fed mice compared with the ND group (*P* < .001). In addition, antioxidant enzyme levels are indicated in Table 5 including MDA, SOD, CAT, and FRAP. A significant decline was seen in SOD, CAT, and FRAP in the group that received HFD for 4 weeks in comparison with the ND group and MDA showed a significant enhancement in the HFD compared with the ND group (*P* < .001).

**Table 4:** Biochemical parameters in the group that received HFD for 4 weeks in comparison to the ND group

Groups	ND group	HFD group
Parameters		
TG (mg/dL)	70.64 ± 2.06	133.25 ± 3.76***
TC (mg/dL)	116.27 ± 3.30	228.50 ± 1.68***
Phospholipid (mg/dL)	245.27 ± 6.19	463.92 ± 6.29***
Total lipid (mg/dL)	243.02 ± 6.08	462.67 ± 7.20***
HDL‐C (mg/dL)	67.72 ± 2.10	30.82 ± 1.24***
LDL‐C (mg/dL)	33.07 ± 2.46	130.55 ± 4.04***
Total protein (g/dL)	2.63 ± 0.13	4.41 ± 0.13***
FBG (mg/dL)	98.75 ± 1.90	124.47 ± 1.16***
Insulin (ng/mL)	0.67 ± 0.06	2.25 ± 0.07***
HOMA	2.90 ± 0.24	12.41 ± 0.29***
Atherogenic index	0.72 ± 0.05	6.55 ± 0.33***

Note

The data are expressed as Mean ± SEM.

Abbreviations: FBG, fast blood glucose; HDL‐C, high‐density lipoprotein cholesterol; HFD, high‐fat diet group; LDL‐C, low‐density lipoprotein‐cholesterol; ND, normal diet group; TC, total cholesterol; TG, triglyceride.

*** *P* < .001 compared with the control group.

**Table 5:** Antioxidant parameters in the group that received HFD for 4 weeks in comparison to the ND group

Groups	ND group	HFD group
Parameters		
MDA (nmol/g)	3.83 ± 0.08	7.09 ± 0.22***
SOD (µ/mg‐p)	19.38 ± 0.72	13.60 ± 0.37***
CAT (µ/mg‐p)	54.23 ± 0.16	41.44 ± 1.23***
FRAP (µmol/mg)	19.52 ± 0.22	13.17 ± 0.33***

Note

The data are expressed as Mean ± SEM.

Abbreviations: CAT, catalase; FRAP, ferric reducing antioxidant power; HFD, high‐fat diet group; MDA, malondialdehyde; ND, normal diet group; SOD: superoxide dismutase.

*** *P* < .001 compared with the control group.

### 3.4 Serum leptin, adiponectin, and TNF‐α evaluation

**Figure 4:**
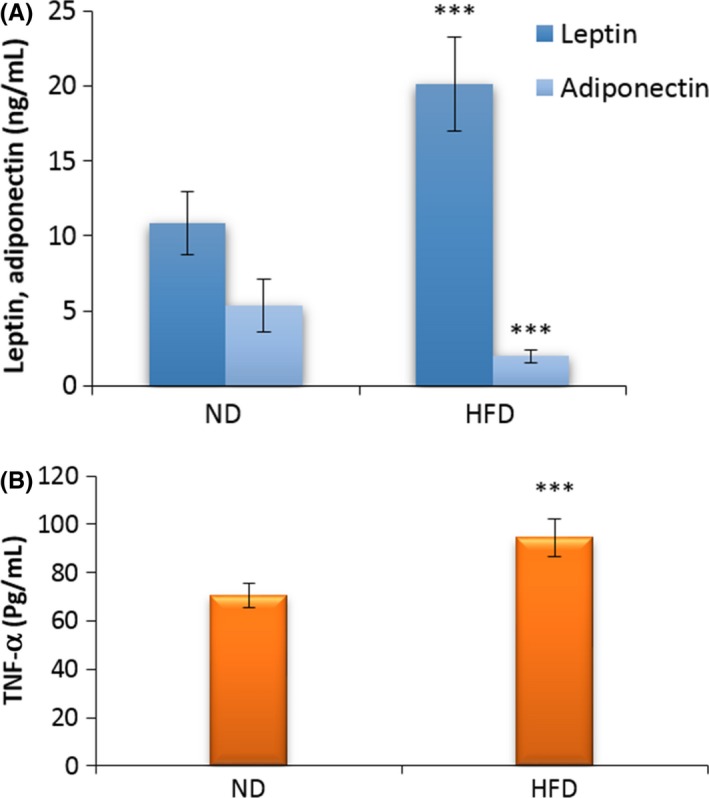
Effect of HFD on serum leptin and adiponectin (A) and TNF‐α (B) in ND and HFD groups at the end of the fourth week of treatment. The data were expressed as means ± SEM. ***P < .001 compared with the control group. HFD, high‐fat diet group; ND, normal diet group

Serum leptin, adiponectin, and TNF‐α values are shown in Figure 4. There was a significant increase in leptin of HFD compared with the ND group. Furthermore, adiponectin level was significantly lower in HFD in comparison with the ND group. TNF‐α level was enhanced significantly in HFD compared with the ND group (*P* < .001).

### 3.5 Liver enzymes evaluation

**Figure 5:**
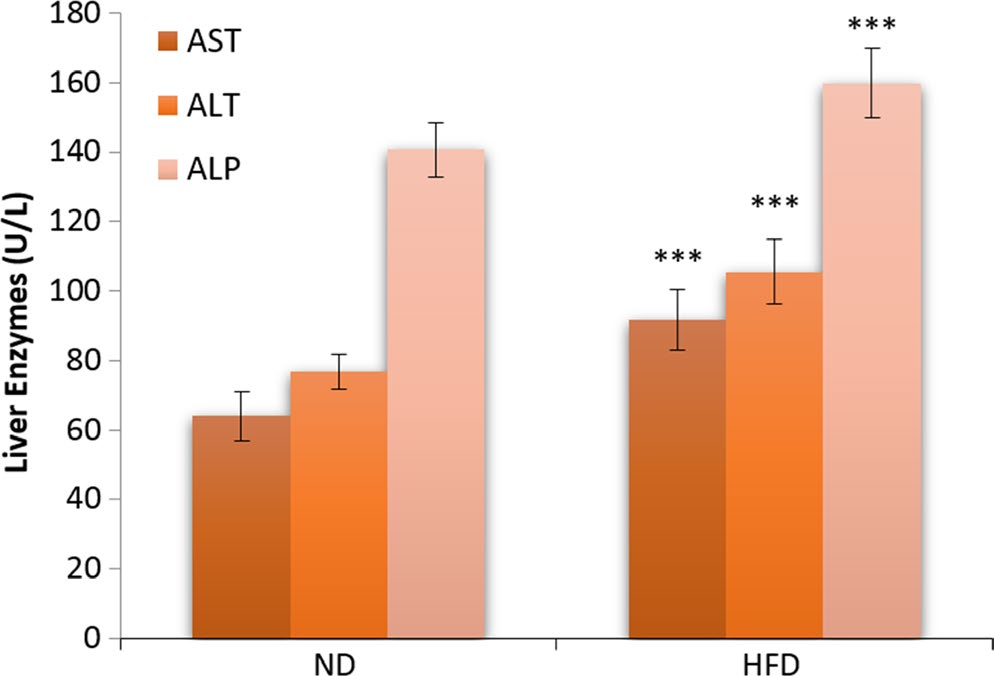
Effect of HFD on serum AST, ALT, and ALP in ND and HFD groups at the end of the fourth week of treatment. The data were expressed as means ± SEM. ***P < .001 compared with the control group. Abbreviations: HFD, high‐fat diet group; ND, normal diet group

Serum AST, ALT, and ALP amounts are shown in Figure 5. There was a significant increase in the serum AST, ALT, and ALP levels in the HFD compared with the ND group at the end of the fourth week of treatment (*P* < .001).

### 3.6 PPAR‐α expression in the liver

**Figure 6:**
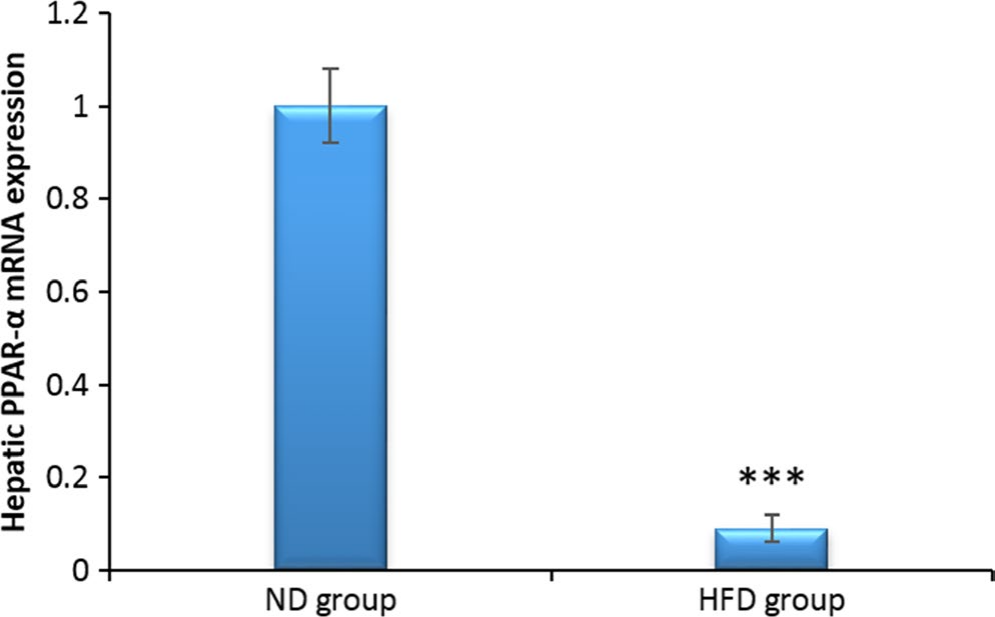
Effect of HFD on PPAR‐α expression in ND and HFD groups at the end of the fourth week of treatment. The data were expressed as means ± SEM. ***P < .001 compared with the control group. Abbreviations: HFD, high‐fat diet group; ND, normal diet group

In this study, the PPAR‐α expression was reduced in the HFD compared with the ND group significantly (Figure 6) (*P* < .001).

## 4 DISCUSSION

This study introduces a new mice model for NASH studies in humans. This new model successfully simulated NASH and fibrosis in 4 weeks. NASH is accompanied with steatosis, serum dyslipidemia, oxidative stress, type 2 diabetes, and insulin resistance.^21^ Our HFD model is composed of components that are found in normal human's dietary, such as sunflower oil, palm oil, and saturated and trans fatty acid‐rich (TFA) oils. The daily and exorbitant consumption of TFA‐rich lipids leads to liver fatty accumulation considerably.^22^

Furthermore, sodium deoxycholate, tween 80, and propylene glycol were used in our HFD model. Sodium deoxycholate is a bile salt that is used as a lipid and phospholipid solubilizer in water and fatty cell solvent^23^ This component facilitates fat absorption in the intestine. Tween 80 is also used as a co‐emulsifier for HFD emulsion.^24^ Eventually, propylene glycol, as an anti‐ketosis, collects blood's free fatty acids (FFAs) that were released by sodium deoxycholate and stored in the liver.^25^

In this research, HFD was administrated simultaneously by intra‐mouth oral gavage and ad libitum feeding. The intra‐mouth gavage of HFD was performed for food intake simulation in human and also for prevention of injury to the esophagus and esophageal sphincter and their inflammation during stomach gavage.

In this model, body weight was increased, but nonsignificantly. This may be due to the elimination of visceral fat through sodium deoxycholate, limiting glucose consumption by insulin resistance that results in energy sources switch to body proteins. The liver weight index, as an aspect of NAFLD and NASH, was significantly enhanced in the HFD compared with the ND group.^26^

In the HFD model, NASH was confirmed by liver histological analysis of tissue slices after 4 weeks of treatment. Fat accumulation, ballooned hepatocytes, micro‐ and macro lipid droplets and hepatocytes with pushed nuclei into the periphery were found in the liver sections of HFD animals. In H&E‐stained livers of the HFD group, some of the important features that characterize NASH were observed, such as bipolar and tripolar cells, small and large droplets, foamy cytoplasm, necrosed cells, MDB, Kupffer cell clusters, and inflammatory cells accumulation, periphery and hyperchromatic nuclei, and ballooned hepatocytes.^27^, ^28^ Kupffer cells and mononuclear inflammatory cell accumulation are involved in the progress of steatohepatitis and fibrosis.^28^ The macrophages have a key role in inflammation and insulin resistance induction; in addition, FFA and cholesterol consumption induce oxidative stress and result into hepatitis, hepatofibrosis, and progression to NASH.^29^ Moreover, in our study, trichrome staining showed fibrosis development.

In our HFD model, the lipid profile parameters including LDL‐C, TG, TC, and phospholipid were increased while HDL‐C declined significantly. In agreement with our study, previous investigations have reported increase of LDL‐C, TG, TC, and phospholipid levels and decrease of HDL‐C in NASH.^30^ In addition, TG is the main fat accumulated in liver, found in both NAFLD and NASH.^31^ The increased TG and decreased HDL levels are strongly related to NAFLD and NASH.^32^ In addition, the TP value was significantly enhanced in our HFD model. A Japanese study has shown that TP level was significantly higher in NAFLD subjects than normal persons.^33^

The FBG, insulin, and HOMA amounts showed a significant increase in our HFD model. Enhanced FBG and insulin levels are prevalent in NAFLD.^33^ Insulin resistance that is determined by HOMA is an important factor in NAFLD pathogenesis.^34^ In parallel with our findings, the severity of steatohepatitis was strongly related to insulin resistance and visceral fat accumulation irrespective of obesity.^33^ In the current study, the atherogenic index amount was increased significantly. HFD administration can induce LDL oxidation in the liver, which is the main factor for atherosclerosis.^35^ A previous study about NAFLD and atherogenic risk factors has proved linkage of atherogenic risk to NAFLD severity. Furthermore, there is a positive link between liver damage and fibrosis and the atherogenic risk.^36^ Human and animal trials have demonstrated that dyslipidemia is a major risk factor for atherosclerosis, and NASH is an acceptable hepatic marker for a cardiovascular risk factor.^28^, ^37^ As a result, our HFD model can be used for atherogenic assessments too.

The antioxidant enzymes, including SOD, CAT, and FRAP showed a significant decrease while MDA has a notable increase in the HFD compared with the ND group. MDA is a sign of oxidative stress and NASH subjects have higher MDA levels compared with normal people.^38^ The electron leakage subsequent to mitochondrial damage is caused by the overproduction of superoxide by SOD activity, while excess superoxide should be eliminated by glutathione peroxidase or catalase enzymes.^39^ Mitochondrial damage disrupts the regulation of lipid metabolism in the liver and stimulate oxidative stress and reactive oxygen species (ROS) leading to peroxidation of fats, extra production of cytokines and apoptosis.^40^ e SOD levels decline in NASH, so this disruption of the antioxidant pathway may play a critical role in NASH pathogenesis.^41^ In previous researches, the SOD and FRAP levels, as antioxidant agents, showed a significant reduction in NAFLD patients in comparison with healthy people .^42^

In the current research, the serum leptin and TNF‐α levels significantly increased, while the adiponectin levels were notably declined. TNF‐α is one of the molecules that play an important role in fatty liver inflammation. TNF‐α affects hepatocytes fat and suppresses adiponectin, while increasing leptin secretion.^39^ TNF‐α, as a pro‐inflammatory agent, increases in response to oxidative stress and contributes to inflammation in the liver via NADPH oxidase induction.^43^ Leptin levels considerably increase in patients with NASH.^44^ Increased leptin levels in NASH lead to disrupting the TNF‐α levels. On the other hand, leptin can induce oxidative stress and subsequent increased production of pro‐inflammatory factors and ultimately leads to hepatosteatosis and fibrosis in NASH.^45^ Adiponectin is a beneficial adipokine that has anti‐diabetes, anti‐inflammation, and anti‐storing lipid properties. Pro‐inflammatory factors such as TNF‐α can repress adiponectin activity. There is a reverse correlation between adiponectin levels and insulin resistance and adiponectin also have an antagonistic effect with leptin in liver inflammatory and fibrosis.^46^, ^47^ Furthermore, HFD consumption decreases adiponectin levels and probably increase NASH progression.^48^ In a previous study, adiponectin levels were found to be low in NAFLD and lower in NASH patients.^46^

In our HFD model, serum AST, ALT, and ALP levels indicated a significant increase in the HFD compared with the ND group at the end of the experiment. Increased ALT is associated with insulin resistance. Elevations of ALT and AST in NAFLD and NASH are related to liver injury. ALT levels are used to determine metabolic syndrome while AST and ALT are used for diagnosis of development to diabetes.^49^ ALT levels are more increased in NASH than simple steatohepatitis. Therewith, enhanced ALT levels are associated with adiponectin, glucose tolerance reduction, and triglyceride and FFAs. Increased AST mostly occurs in patients with enhanced ALT levels and is involved in liver diseases or cirrhosis. ALP levels are usually more increased in persons with NASH and fibrosis than people without fibrosis.^50^

In our study, PPAR‐α expression was significantly reduced in the HFD compared with the ND group. PPAR‐α is the major isotype in the liver and plays the main role in fat metabolism during starvation.^51^ Furthermore, it is known that PPAR‐α mediates hepatic genes expression that modulates lipid metabolism in response to fat diet consumption. PPAR‐α is a main regulator of fat metabolism in the liver and acts by regulating the expression of many genes involved in the peroxisomal and mitochondrial oxidation of fats, inflammation factors, and metabolism of glucose.^52^ A previous study has reported that PPAR‐α modulates acute‐phase response signaling and inflammation factor's secretion in the rodent models with atherosclerosis, systematic inflammation, and NASH.^53^ Finally, PPAR‐α has a major role in NAFLD and NASH, so that its elimination or low expression leads to steatosis and steatohepatitis.^51^ Current results showed that our HFD model successfully simulated all aspects of NASH with fibrosis in 4 weeks. This HFD model has reproduced all parameters that change in NASH and may be used as an atherosclerosis model too. Furthermore, the current HFD model is inexpensive and quickly obtained.

## CONFLICT OF INTEREST

None.
